# Genetic basis for phenotypic differences between different *Toxoplasma gondii* type I strains

**DOI:** 10.1186/1471-2164-14-467

**Published:** 2013-07-10

**Authors:** Ninghan Yang, Andrew Farrell, Wendy Niedelman, Mariane Melo, Diana Lu, Lindsay Julien, Gabor T Marth, Marc-Jan Gubbels, Jeroen PJ Saeij

**Affiliations:** 1 Biology Department, Massachusetts Institute of Technology, 77 Massachusetts Ave, building 68-270, Cambridge, MA 02139, USA; 2 Department of Biology, Boston College, 140 Commonwealth Avenue, Chestnut Hill, Boston, MA 02467, USA

**Keywords:** *Toxoplasma*, Type I strains, Comparative genomics, Transcriptomics, NF-ĸB

## Abstract

**Background:**

*Toxoplasma gondii* has a largely clonal population in North America and Europe, with types I, II and III clonal lineages accounting for the majority of strains isolated from patients. RH, a particular type I strain, is most frequently used to characterize *Toxoplasma* biology. However, compared to other type I strains, RH has unique characteristics such as faster growth, increased extracellular survival rate and inability to form orally infectious cysts. Thus, to identify candidate genes that could account for these parasite phenotypic differences, we determined genetic differences and differential parasite gene expression between RH and another type I strain, GT1. Moreover, as differences in host cell modulation could affect *Toxoplasma* replication in the host, we determined differentially modulated host processes among the type I strains through host transcriptional profiling.

**Results:**

Through whole genome sequencing, we identified 1,394 single nucleotide polymorphisms (SNPs) and insertions/deletions (indels) between RH and GT1. These SNPs/indels together with parasite gene expression differences between RH and GT1 were used to identify candidate genes that could account for type I phenotypic differences. A polymorphism in dense granule protein, GRA2, determined RH and GT1 differences in the evasion of the interferon gamma response. In addition, host transcriptional profiling identified that genes regulated by NF-ĸB, such as interleukin (IL)-12p40, were differentially modulated by the different type I strains. We subsequently showed that this difference in NF-ĸB activation was due to polymorphisms in GRA15. Furthermore, we observed that RH, but not other type I strains, recruited phosphorylated IĸBα (a component of the NF-ĸB complex) to the parasitophorous vacuole membrane and this recruitment of p- IĸBα was partially dependent on GRA2.

**Conclusions:**

We identified candidate parasite genes that could be responsible for phenotypic variation among the type I strains through comparative genomics and transcriptomics. We also identified differentially modulated host pathways among the type I strains, and these can serve as a guideline for future studies in examining the phenotypic differences among type I strains.

## Background

*Toxoplasma gondii* is an obligate intracellular pathogen from the phylum Apicomplexa and is estimated to infect about one third of the world population [1]. It usually establishes an asymptomatic, chronic infection, but immunodeficient individuals can develop severe disease such as encephalitis and retinitis. *Toxoplasma* has a relatively complex life cycle, containing both asexual and sexual stages. The sexual cycle occurs in felines, but because a single parasite can give rise to both micro and macro-gametes, usually self fertilization occurs [2]. Sexual recombination leading to new genotypes can only occur when felines are infected simultaneously with at least two different strains [3]. The low occurrence of concurrent infection events, together with horizontal transmission between intermediate hosts through consumption of infectious cysts, likely accounts for the highly clonal population structure observed in North America and Europe [4].

In North America, three clonal lineages known as types I, II and III were thought to predominate [5,6]. However, recent SNP analysis at five loci in ~950 strains, representing worldwide diversity, clustered these strains into 15 haplogroups and showed a high prevalence of type 12 strains in North America [7], while a large number of divergent strains are present in South America. Using genome-wide SNPs, it was shown that even within these haplogroups (except for haplogroups I, II, III and 6), there is often significant diversity and most strains appear to have formed through recent recombination events [8]. Between lineage diversity is estimated to be 1 to 3% while diversity within the type I, II and III clonal lineage is ~0.01% [8-10]. There are also strong phenotypic differences between lineages [11], such as acute virulence in mice, with type I strains being uniformly lethal (LD_100_ = 1), while types II and III are less virulent, with LD_50_ ≥ 10^3^ and LD_50_ ≥ 10^5^, respectively [11]. Another phenotype that has been correlated with virulence is *in vitro* growth rate, with type I parasites having a higher growth rate compared to types II and III [12].

RH is the most commonly used type I strain for characterization of numerous aspects of *Toxoplasma* biology, ranging from active invasion, replication, to host cell egress, and it has been used extensively for molecular genetic analyses. RH was initially isolated from a 1939 case of toxoplasmic encephalitis by Albert Sabin and was subsequently cloned by Elmer Pfefferkorn for *in vitro* culture in 1977 [13,14]. This cloned RH-ERP strain has likely undergone laboratory adaptation due to extensive *in vitro* lab passage, unlike RH-JSR, a non-cloned RH isolate that was propagated in mice and has undergone little serial passage in culture [15]. There are several other isolates of the type I lineage defined by PCR-RFLP at multiple marker alleles, such as GT1, an isolate from goat skeletal muscle [16], from which the complete genome has been sequenced (ToxoDB.org) [17].

Surprisingly, RH-ERP displays significant parasite phenotypic variation compared to GT1 and RH-JSR [15], with RH-ERP having increased extracellular survival times, higher *in vitro* growth rates and loss of ability to form orally infective cysts [15,18]. Moreover, using *Toxoplasma* microarrays, it was observed that RH-ERP parasite gene expression is significantly different compared to RH-JSR and GT1 [15], with upregulation of certain ABC transporters in RH-ERP compared to RH-JSR or GT1. RH-ERP and GT1 also differ in the modulation of certain host processes; a serum response factor (SRF) reporter cell line is activated more by RH-ERP compared to GT1, and transcript levels of early growth response factor 2 (EGR2), a SRF target gene, are higher upon RH-ERP infection compared to GT1 [19,20]. In addition, there is increased immune related GTPase (IRG) coating of the parasitophorous vacuole membrane (PVM) of GT1 compared to RH-ERP in IFN-γ stimulated mouse embryonic fibroblasts (MEFs) and subsequent higher levels of GT1 killing compared to RH-ERP [21]. Differential modulation of host pathways could affect host cell survival or nutritional availability and subsequent *Toxoplasma* replication within the host cell.

The genetic basis for phenotypic variation between RH-ERP and the other type I strains is currently unknown. Many of these phenotypic differences are important determinants of acute virulence and chronic infection, as increased growth rates can lead to higher parasite burdens *in vivo*, and cyst formation is essential for *Toxoplasma* transmission [1]. Thus, understanding the genetic basis for the enhanced growth rate of RH-ERP and its inability to form orally infectious cysts could provide important insights into *Toxoplasma* virulence determinants. To examine the potential genetic basis for phenotypic differences between RH-ERP and GT1, we compared the complete genome sequences of RH-ERP and GT1 and identified single nucleotide polymorphisms (SNPs) and insertions/deletions (indels) across the genome. We also compared differential parasite gene expression between RH-ERP and RH-JSR/GT1 through our own independent transcriptional profiling and previous studies [15,22]. Using our SNP/indel and differentially expressed gene list, we then identified a number of candidate genes that could be responsible for the phenotypic differences observed between RH-ERP and RH-JSR/GT1, including a dense granule protein, GRA2. We then attributed the differences between RH-ERP and GT1 in evasion of IFN-γ-mediated killing in MEFs to GRA2. To identify novel differences in host cell modulation among type I strains, we performed transcriptional profiling of human foreskin fibroblasts (HFFs) infected with RH-ERP, RH-JSR and GT1. Several host pathways were found to be differentially modulated, such as the NF-κB pathway, which is activated by GT1 but not by RH-ERP or RH-JSR. Other host phenotypes that were different across type I strains included IL-12p40 (a NF-κB dependent cytokine) secretion by infected macrophages and recruitment of p-IκBα to the PVM, which was partially dependent on GRA2. Our results show that genetic mutations accumulated over time due to continuous laboratory passaging, can lead to large phenotypic changes and the candidate genes identified can serve as a guideline for future studies in examining phenotypic differences among type I strains.

## Methods

### Parasites and cells

Human foreskin fibroblasts (HFFs) were grown in DMEM (Invitrogen) supplemented with 10% heat inactivated FBS (PAA), 50 μg/ml each of penicillin and streptomycin, and 20 μg/ml gentamycin. A HEK293T stable reporter cell line with four copies of the NF-κB consensus transcriptional response element driving the expression of GFP and luciferase (System Biosciences) were grown in the same DMEM but supplemented with 1 mM sodium pyruvate, 100 μM MEM nonessential amino acids and 10 mM HEPES. These NF-κB 293T reporter cells were passed every 2–4 days using 0.05% trypsin-EDTA [23]. C57BL/6J mouse embryonic fibroblasts (MEFs) were a gift from A. Sinai (University of Kentucky College of Medicine, Lexington, KY) and grown in HFF media supplemented with 10 mM HEPES. Parasites were maintained *in vitro* by serial passage on monolayers of HFFs at 37°C in 5% CO_2_. RH-ERP is a clone of the original RH isolate, subjected to continuous passage *in vitro* until the time of present study. RH-JSR was a gift from David Sibley (Washington University in St. Louis, Saint Louis, MO) and is a noncloned line of the original RH isolate, subjected to propagation in mice and cryopreserved since 1988. GT1 was originally isolated from skeletal muscle of a goat in 1980, and was subject to passage in laboratory conditions [15].

### Reagents

All tissue culture reagents were purchased from Gibco (Life Technologies Corporation, USA), unless otherwise stated. Antibodies against *Toxoplasma* dense granule protein GRA7 were kindly provided by John Boothroyd (Stanford University, Palo Alto, CA) [24]. Anti-mouse p-IκBα (sc-8404), anti-human NF-κB p65 (sc-109) and anti-mouse TGTP (sc-11079) antibodies were purchased from Santa Cruz Biotechnology (California, USA). Recombinant human TNFα was obtained from Invitrogen (Life Technologies Corporation, USA), and lipopolysaccharide was purchased from EMD Millipore (Merck KGaA, Darmstadt, Germany).

### Generation of transgenic parasites

To generate RH-ERP (∆HXGPRT) parasites expressing the GT1 allele of GRA15, the GRA15 coding region and putative promoter (1,940 bp upstream of the start codon) was amplified from GT1 genomic DNA by PCR (forward, 5′–CACCTTGACTGCCACGTGTAGTATCC–3′, reverse, 5′-TTA*CGCGTAGTCCGGGACGTCGTACGGGTA*TGGAGTTACCGCTGATTGTGT–3′). Sequence coding for a C terminal HA tag was included in the reverse primer (denoted with italics). GRA15_GT1_HA was then cloned into pENTRD/D-TOPO (Invitrogen), and into pTKO-att [23] through LR recombination (Invitrogen). The pTKO-att-GRA15_GT1_HA vector was then linearized by digestion with XhoI (New England Biolabs, Inc.). Linearized vector was transfected into RH-ERP∆HXGPRT by electroporation as previously described [23]. Stable integrants were selected in media with 50 μg/ml mycophenolic acid (Axxora) and 50 μg/ml xanthine (Alfa Aesar) and cloned by limiting dilution. Immunofluorescence was used to confirm expression of GRA15_GT1_ via HA staining.

To generate RH∆*gra2* parasites complemented with either RH-ERP GRA2 or GT1 GRA2, the GRA2 coding region and putative promoter (1,143 bp upstream of the start codon) was amplified from RH-ERP (∆HXGPRT) and GT1 genomic DNA by PCR (forward, 5′-**GGGGACAACTTTTCTATACAAAGT**TGAGCATGTAGGTGGAACGC-3′, reverse, *5*′-TTA*CGCGTAGTCCGGGACGTCGTA*CGGGTACTGCGAAAAGTCTGGGAC-*3*′). Sequence coding for the attP4r recombination site was included in the forward primer and a C terminal HA tag was included in the reverse primer (attP4r in bold and HA tag in italics). A second PCR added the attP3 recombination site after the HA tag and the insert was cloned into pDONR 221 P4r-P3r (Invitrogen) using BP recombination (Invitrogen). The GRA2-HA insert was then flanked by the genomic DNA both upstream and downstream of the UPRT locus and inserted into the pTKO2 destination vector [23] by LR recombination (Invitrogen). pTKO2-GRA2HA was linearized by digestion with HindIII (New England Biolabs, Inc.) which does not cut within GRA2 and pTUB-CAT was digested with NotI (New England Biolabs, Inc.). pTKO2-GRA2HA and pTUB-CAT were co-transfected into RH∆*gra2* as previously described [23]. Stable integrants were selected by passage of 10^6^ parasites every 2 days in 2 μM chloramphenicol and cloned by limiting dilution. Immunofluorescence and Western blot were used to confirm expression of RH-ERP GRA2 or GT1 GRA2 via HA staining.

### Luciferase assays

HEK293T NF-κB reporter cells were seeded at a density of 4 × 10^4^ cells per well for 4 hours in a black 96 well clear bottom plate (Corning). Parasites were syringed lysed, washed once with PBS and three different multiplicity of infection (MOIs) per strain were used to infect reporter cells. As a positive control, recombinant human TNFα was used at 20 ng/μl to stimulate uninfected cells at the same time as infection. After 24 hours of infection, uninfected, stimulated and infected cells were lysed using Cell Culture Lysis reagent, and luciferase activity in lysates was measured according to manufacturer’s protocol (Promega). Data from cells infected with similar MOIs, as determined by plaque assay, were used.

### In vitro cytokine ELISAs

C57BL/6 bone marrow-derived macrophages (BMMs) were isolated as described [25], and plated in DMEM, supplemented with 20% L929 supernatants, two days before infection. Parasites were syringe lysed, washed once with PBS and three different MOIs per strain were used to infect uninfected macrophages. As a positive control, purified lipopolysaccharide (100 ng/mL) was used to stimulate uninfected BMMs 3 hours before supernatants were collected. After 24 hours of infection, supernatants from uninfected, stimulated and infected cells were collected and stored at −80°C until ELISAs were performed. IL-12/23p40 and CCL2/MCP-1 levels in culture supernatants were determined using commercially available ELISA kits (ELISA DuoSet®, R&D Biosystems), according to the manufacturer’s instructions.

### Immunofluorescence

Irgb6 staining in MEFs were performed as previously described [21]. Percent Irgb6 coating was determined in a blind fashion by finding intracellular parasites and then scoring Irgb6 coating as positive or negative. For p65 and p-IκBα staining, HFFs were plated on coverslips in 24 well plates until confluent, and subsequently infected with parasites for different timepoints. HFFs were then fixed with 3% (vol/vol) formaldehyde in PBS for 20 min at room temperature, permeabilized with 0.2% (vol/vol) Triton X-100 in PBS, and blocked in PBS with 3% (wt/vol) BSA and 5% (vol/vol) goat serum. Coverslips were incubated with primary antibodies overnight at 4°C, and fluorescent secondary antibodies, coupled with Alexa Fluor 488 or 594 (Invitrogen), and Hoechst dye were used for antigen and DNA visualization, respectively. Coverslips were mounted on a glass slide with Vectashield (Vector laboratories) and photographs were taken using NIS-Elements software (Nikon) and a digital camera (CoolSNAP EZ, Roper industries) connected to an inverted fluorescence microscope (model eclipse Ti-S, Nikon).

Quantification of p65 nuclear localization was performed as previously described [23]. Quantification of p-IκBα localized to the PVM was performed using NIS-Elements software (Nikon). Parasitophorous vacuoles (PVs) were chosen at random through GRA7 staining. The intensity of fluorescent p- IκBα was measured by drawing two lines at right angles across the long and short axes of the vacuoles, and intensity profiles were obtained for each line. The fold change intensity of each line was taken by dividing the highest peak value where each line crossed the margins of the PVM by the lowest value for each line (taken as background). The signal intensity for each vacuole was given as the mean of the two intensity fold changes obtained per vacuole.

### Plaque assays

Plaque assays were set up as previously described [21]. Briefly, MEFs were seeded the day before, and stimulated with 1000 U/ml mouse IFNγ or left unstimulated before infection in a 24 well plate. Plaques were then counted in unstimulated and stimulated MEFs after incubation for 4–7 days at 37°C.

### SNP and INDEL list generation between RH-ERP and GT1

To reduce the posibility of sequence errors and cell line specific mutations, genomic DNA from two related RH-ERP lines, 2F-1-YFP2 parent and an F-P2 ENU mutagenized child, were prepared and sequenced [26]. Illumina sequencing produced 40,495,290 and 43,514,016 reads for the parent and F-P2 samples respectively. The FASTQ sequence traces were aligned to a FASTA reference containing both *Toxoplasma gondii* GT1 genomic reference v5.0 and the Human genome reference build 37. Reads were aligned to the GT1 genome as well as the Human genome to filter out any possible human contamination. MOSAIK v1.0 was used to perform the alignments using the standard parameters. Greater than 90% of the reads aligned for each sample, with rougly half of the reads filtered out as human contamination. Variants were called using the variant caller FreeBayes [27] using standard parameters, software version 0.7.2. Varations were then filtered to identify variants that were called in both parental and F-P2 samples at an allele balance greater than 75%.

### RH-ERP and GT1 gene expression via RNA-seq

RNA was isolated from C57BL/6 bone marrow derived macrophages infected with RH-ERP and GT1. These were processed and sequenced as described previously [8]. Reads were then mapped to the GT1 genome (ToxoDB.org).

### *Toxoplasma* microarray analysis

For *Toxoplasma* arrays, image analysis files (.CEL) files from published microarray data from HFFs infected with RH-ERP, RH-JSR or GT1 were downloaded from the Gene Expression Omnibus (GEO) database (Series GSE16115 and GSE22315). In addition, independent *Toxoplasma* arrays were performed on total RNA isolated from HFFs infected for 24 hours with RH-ERP, RH-JSR and GT1 (Series GSE44191). Each sample was hybridized to the *T*. *gondii* Affymetrix microarray [22]. The image analysis files from GSE16115, GSE22315 and GSE44191 were all processed together using the Expression Console Software, normalized using Robust Multi Array (RMA) algorithm, and all background values less than 6.5 were set to 6.5. These arrays were then imported into Multi-Experiment Viewer (MeV) [28], all genes were median-centered and loaded into Genomica [29] as the array dataset.

To create Genomica and GSEA custom gene sets to determine biological function enrichment [29,30], we downloaded the Gene Ontology (GO) and InterPro (IP) annotations for *Toxoplasma* genes using the ME49 v8 reference genome (ToxoDB.org). To determine functional enrichment in groups of genes with similar annotations (gene sets), gene sets were loaded into Genomica [29] and selected as gene sets to analyze. The default parameters were used to run the hypergeometric enrichment analysis (the complete set of *Toxoplasma* gene IDs and their associated functional assignments in Genomica or GSEA format is available from the authors upon request).

### Human microarray analysis

HFFs were grown in 6 well plates until confluency was reached. Parasites were syringe lysed and washed once with PBS. HFFs were infected with RH-ERP, RH-JSR and GT1 at three different MOIs for 24 hours. Plaque assays were done to assess viability of parasites and infections with similar MOIs were chosen. Three biological replicates were done for RH-ERP and RH-JSR while two biological replicates were done for GT1. TRIzol reagent (Invitrogen) was used to isolate total RNA according to the manufacturer’s instructions and RNA was cleaned up using MiniElute kit (Qiagen). RNA was labeled and hybridized to human Affymetrix arrays (Human U133A 2.0) according to manufacturer’s protocol. Probe intensities were measured with the Affymetrix GeneChip Scanner 3000 7G and were processed into image analysis (.CEL) files with Expression Console Software (Affymetrix), which can be accessed through GEO (GSE44189). Intensity values were normalized using RMA through Expression Console, and all background values less than 6.5 were set to 6.5.

GSEA was used to find candidate transcription factors and canonical pathways that were modulated differently between the type I *Toxoplasma* strains [30]. Both transcription factor and canonical pathway gene sets from the Molecular Signatures Database (MSigDB) were used to determine enrichment (c2.cp. symbols, c3.tft. symbols), using default parameters except the range of set size, which was changed to a minimum of 5 and maximum of 3000. Analysis of distant regulatory elements of coregulated genes (DiRE, http://dire.dcode.org) [31] was performed using a random set of 5000 background genes and using target elements of top 3 evolutionary conserved regions (ECRs) and promoter ECRs. For every gene in a list, DiRE detects regulatory elements throughout the entire gene locus and looks for enrichment of transcription factor binding sites.

## Results and discussion

### Identification of genetic differences between RH-ERP and GT1

To determine the genetic differences between RH-ERP and GT1, we sequenced RH-ERP using Illumina sequencing and downloaded the complete genome sequence of GT1 (Table 1A and ToxoDB.org). A list of single nucleotide polymorphisms (SNPs) and insertions/deletions (indels) between RH-ERP and GT1 was generated, and a total number of 1,394 SNPs and indels were identified. There were 230 SNPs/indels within predicted coding regions, 484 SNPs/indels within predicted genes but outside coding regions, and 680 SNPs/indels outside predicted genes. From the 230 SNP/indels within predicted coding regions, we further identified 133 SNPs leading to nonsynonymous amino acid changes, 43 SNPs leading to synonymous amino acid substitutions, and 54 indels within predicted coding regions (Table 1B and Additional file 1: Table S1).

**Table 1:** Genetic differences between RH-ERP and GT1

**A. Summary of comparative genome sequencing between RH-ERP and GT1**						
Description			RH-ERP (parental)		RH-ERP F-P2 (mutant)	
Total base pairs sequenced			6.1 × 10^9^		6.5 × 10^9^	
Total paired end reads			4.0 × 10^7^		4.4 × 10^7^	
Total reads aligned			3.7 × 10^7^		3.9 × 10^7^	
Total reads aligned (%)			90.1		90.5	
Human reads (%)			32.2		49.4	
*Toxoplasma gondii* reads (%)			57.9		44.1	
GT1 genome covered (%)			96.5		96.9	
Reads in unassembled contigs (%)			0.9		0.7	
Total Shared Variation Called			1,394			
**B. Genetic differences between RH-ERP and GT1**						
SNPs/indels within predicted coding regions		230	Nonsynonymous SNPs		133	
			Synonymous SNPs		43	
			Indels		54	
SNPs/indels within predicted genes, outside coding regions		484	SNPs/indels in 1000 bp upstream of predicted ATG start (5′UTR)		SNPs/indels in 1000 bp downstream of predicted end codon (3′UTR)	
			133		143	
SNPs/indels outside predicted genes		680				
**C. Functional enrichment in genetic differences between RH-ERP and GT1**						
Gene set	Enrichment in Gene Ontology function	P-value	Number of annotated genes with SNPs/indels	Percent of annotated genes with SNPs/indels in gene set (%)	Total number of genes annotated	Percent of annotated genes in genome (%)
Nonsynonymous SNPs and indels within predicted gene coding regions	3′5′-cyclic nucleotide phosphodiesterase	3.57^-03^	3	2.19	16	0.24
	Protein kinase	9.74^-03^	7	5.11	120	1.75
	ATP binding	0.02	11	8.03	270	3.93
SNPs/indels within 5′ UTR of predicted genes (1000 bp upstream of ATG start)	Nucleoside triphosphatase	8.47^-03^	4	4.17	58	0.85
	Nucleotide binding	0.01	5	5.21	103	1.50
	ATPase activity	0.03	3	3.13	48	0.70

**(A)** Details for genome sequencing of RH-ERP parental and RH-ERP F-P2 mutant, where common variants between these two strains were determined as SNPs/indels between RH-ERP and GT1. **(B)** Total number of single nucleotide polymorphisms (SNPs) and insertions/deletions (indels) identified between RH-ERP and GT1, with the number of SNPs/indels identified in each category as indicated. **(C)** Top three functional enrichment of nonsynonymous SNPs and indels present in gene coding regions, together with genes which contain SNP/indels 1000 bp upstream of predicted ATG start, using hypergeometric enrichment.

We then determined whether there was functional enrichment in the genes containing the 1,394 SNPs/indels. Genes with nonsynonymous SNPs and indels within coding regions were enriched in 3′5′ cyclic nucleotide phosphodiesterase, protein kinase, ATP binding and metal ion binding activities (*p*-value < 0.05). Genes with SNP/indels within 1000 bp upstream of predicted ATG start were enriched in nucleoside triphosphatase, nucleotide binding and ATPase activities (*p*-value < 0.05) (Table 1C). Interestingly, addition of a cGMP-specific phosphodiesterase inhibitor, Zaprinast, has been shown to induce *Toxoplasma* egress from host cells [32]. Moreover, deletion of phosphodiesterase δ in a related Apicomplexan, *Plasmodium berghei*, caused severe defects in formation of normal ookinetes and ookinete gliding motility [33]. Thus, it is possible that the phosphodiesterases that have nonsynonymous SNPs and/or indels could contribute to differences in growth rate of RH-ERP compared to GT1.

RH-ERP tachyzoites grow faster, have higher extracellular viability and loss in ability to form orally infectious cysts compared to GT1/RH-JSR tachyzoites [15,18]. Thus, these phenotypic differences are likely due to parasite genes that are polymorphic and/or differentially expressed between RH-ERP and GT1. We used our SNP/indel data and *Toxoplasma* gene expression data to identify candidate genes, focusing on genes that have non-synonymous, non-conservative SNPs and/or are differentially expressed between RH-ERP and RH-JSR/GT1.

### Analysis of genes with polymorphisms in coding regions between RH-ERP and GT1

From the list of 133 nonsynonymous SNPs, we identified 104 SNPs which led to non-conservative amino acid changes. We then identified 33 SNPs in genes which had transcript levels expressed above background levels of 6.5 in RH-ERP tachyzoites through *Toxoplasma* array data [15,22], and identified several candidate genes which could lead to differences in phenotypes between RH-ERP and GT1 (Table 2A and Additional file 1: Table S1). The gene encoding for dense granule protein GRA2 (TGGT1_083030) was found to have a glycine (GT1) to serine (RH-ERP) substitution, and was expressed highly in both RH-ERP and GT1 from array data. RHΔ*gra2* differs in several phenotypes compared to parental RH-ERP (∆HXGPRT), such as the disruption of the intravacuolar network within the PV, decreased virulence in mice and enhanced susceptibility to IRG-mediated killing [21,34,35].

**Table 2:** Top candidate genes that have nonsynonymous amino acid changes between RH-ERP and GT1

**A. Candidate genes containing nonsynonymous SNPs between RH-ERP and GT1**				
ToxoDB GT1 ID	ToxoDB annotation	A.A. change (GT1 to RH-ERP)	RH-ERP expression (Microarray)	Possible involvement in *Toxoplasma* process
TGGT1_083030	28 kDa antigen, putative (GRA2)	Gly to Ser	13.8	Growth
TGGT1_113990	SRS29C	Ser to Phe	13.5	Invasion
TGGT1_114020	SRS29A	Val to Met	12.7	Invasion
TGGT1_069190	DEAD/DEAH box helicase, putative	Ile to Asn	11.9	Transcriptional control
TGGT1_030300	acid phosphatase, putative (GAP50)	Ile to Asn	11.4	Gliding motility
TGGT1_020630	conserved hypothetical protein (contains glycosyltransferase 17 family)	Leu to Arg	11.1	Growth
TGGT1_066370	hypothetical protein (eukaryotic initiation factor 3)	Leu to Phe	10.7	Translation
TGGT1_081400	ATP-dependent RNA helicase, putative	Arg to Cys Ala to Asp	9.6	Transcriptional control
TGGT1_069890	apoptosis-regulating basic protein, putative	Leu to Phe	8.8	Growth
TGGT1_118630	U2 small nuclear ribonucleoprotein, putative	Thr to Ile	8.7	Growth
TGGT1_098160	Coronin, putative	Met to Val	8.6	Gliding motility
TGGT1_086050	sushi domain-containing protein (RON1)	Asp to Gly	7.9	Invasion
TGGT1_009970	CCR4-NOT transcription complex subunit, putative	Pro to Ser	7.6	Transcriptional control
**B. Candidate extracellular viability genes containing nonsynonymous SNPs between RH-ERP and GT1**				
ToxoDB GT1 ID	ToxoDB annotation	A.A. change (GT1 to RH-ERP)	Fold change between extracellular and intracellular tachyzoites	
TGGT1_057870	conserved hypothetical protein (contains RNA recognition motif)	Asp to His	2.6	
TGGT1_021310	pinA, putative (contains forkhead associated domain)	Phe to Leu	2.0	
TGGT1_104520	conserved hypothetical protein	Leu to Pro	2.0	
TGGT1_016250	hypothetical protein (cyclic nucleotide-binding domain-containing protein)	Ile to Asn	2.0	
TGGT1_065470	conserved hypothetical protein (contains oligomerization domain)	Ser to Cys	-2.3	
TGGT1_098520	EF hand domain-containing protein, putative	Val to Phe	-2.6	
TGGT1_087130	apyrase, putative	Pro to Leu	-3.2	
TGGT1_026200	acylamino-acid-releasing enzyme, putative	Trp to STOP	-3.5	
**C. Candidate bradyzoite genes containing nonsynonymous SNPs between RH-ERP and GT1**				
ToxoDB GT1 ID	ToxoDB annotation	A.A. change (GT1 to RH-ERP)	Fold change between 8 day M4 *in vitro* bradyzoites and 2 day M4 tachyzoites	Fold change between 21 day M4 *in vivo* cysts and 2 day M4 tachyzoites
TGGT1_013440	conserved hypothetical protein	Gly to Ser	1.8	1.6
TGGT1_020630	conserved hypothetical protein (contains glycosyltransferase 17 family)	Leu to Arg	2.3	1.5

**(A)** Candidate genes that have non-conservative, nonsynonymous amino acid changes between RH-ERP and GT1 and are expressed highly in RH-ERP as determined by *Toxoplasma* arrays. *Toxoplasma* array expression values are log2 transformed values, ranging from 6.5 (minimum) to 14.3 (maximum). **(B)** Candidate genes that are differentially expressed between intracellular and extracellular RH-ERP tachyzoites [44]. **(C)** Candidate genes that are differentially expressed between M4 *in vitro* tachyzoites, *in vitro* induced bradyzoites and *in vivo* cysts [46].

Interestingly, an ATP-dependent RNA helicase (TGGT1_081400) contained one conservative and two non-conservative nonsynonymous SNPs, indicating possible positive selection in this gene and was highly expressed in RH-ERP and GT1 from array and RNAseq data (Additional file 1: Table S1). RNA helicases of the DEAD box family are involved in multiple aspects of RNA metabolism, ranging from formation of the exon junction complex, mRNA export and translation initiation [36]. Interestingly, eIF4A, the model of DEAD helicases, has been shown to be downregulated at the transcript level in attenuated type I tachyzoites (through prolonged *in vitro* passage) and type II bradyzoites compared to virulent type I tachyzoites [37].

In addition, SRS29A and SRS29C both had a non-conservative, nonsynonymous amino acid substitution, and were highly expressed in both RH-ERP and GT1 in array data. SRS29A was also 1.7 fold more highly expressed in RH-ERP extracellular tachyzoites compared to RH-ERP intracellular tachyzoites (Additional file 1: Table S1). A recent study showed that RH-ERP overexpressing SRS29C was significantly attenuated in mouse virulence compared to parental RH-ERP strain, though this overexpressing strain did not have any significant differences from the parental strain with regards to invasion, attachment or growth *in vitro*[38].

Another candidate gene identified was GAP50 (TGGT1_030300), with a isoleucine (GT1) to asparagine (RH-ERP) substitution and was highly expressed in both RH-ERP and GT1 in array data (Table 2A). GAP50 is the membrane anchor of the glideosome complex, which is required for gliding motility [39]. It also interacts with other components such as TgMyoA, TgMLC1 and TgGAP45, and requires N-glycosylation for proper localization to the inner membrane complex [40,41]. Interestingly, a rhoptry neck protein, RON1 (TGGT1_086050), has an aspartic acid (GT1) to glycine (RH-ERP) substitution, and is 1.3 fold more highly expressed in RH-ERP compared to GT1 in RNAseq data (Additional file 1: Table S1). RON1 has a distinct rhoptry neck localization [42], and several rhoptry neck proteins constitute the moving junction, which is required for invasion of the host cell [43]. Thus, polymorphisms in these genes could account for differences in invasion, growth or extracellular viability between RH-ERP and GT1.

We also examined these 104 non-conserved, nonsynonymous SNPs for genes that are differentially expressed between RH-ERP intracellular and extracellular tachyzoites [44], as RH-ERP has increased extracellular viability compared to GT1 [15]. We noted a predicted acylamino-acid releasing enzyme (TGGT1_026200) that contained a nonsense mutation leading to a premature stop codon, and had decreased expression in extracellular tachyzoites (Table 2B). In addition, a putative pinA gene (TGGT1_021310) with a phenylalanine (GT1) to leucine (RH-ERP) substitution had increased 2.0 fold expression in extracellular tachyzoites compared to intracellular tachyzoites. This parasite gene contains a forkhead associated domain that is involved in binding to phosphopeptides and forkhead-type transcription factors are involved in the regulation of cell cycle stage specific transcription in budding yeast [45]. Thus, these genes could be involved in the difference in extracellular viability reported between RH-ERP and GT1.

RH-ERP also exhibits a loss in ability to form orally infective cysts, unlike GT1 [18]. Thus, we also examined the 104 non-conserved, nonsynonymous SNPs for parasite genes which are differentially expressed between M4 (type II) tachyzoites, M4 *in vitro* bradyzoites and M4 *in vivo* cysts [46]. Two parasite genes containing substitution polymorphisms between RH-ERP and GT1 were upregulated in both *in vitro* bradyzoites and *in vivo* cysts (Table 2C). One of the genes (TGGT1_020630) contains a glycosyltransferase domain that is involved in transferring N-acetylglucosamine to the core mannose of complex N-glycans. Interestingly, TGGT1_020630 was also identified to be consistently more highly expressed in RH-ERP compared to GT1 (Table 3B). Thus, this gene could potentially be involved in the loss of ability in RH-ERP to form orally infectious cysts, as bradyzoites contain numerous amylopectin granules and the tissue cyst wall consists of lectin binding sugars [47,48].

**Table 3:** Top candidate genes that are differentially expressed between RH-ERP and GT1

**A. Candidate genes containing 5′ and 3′ UTR SNP/indels and are differentially expressed between RH-ERP and GT1**				
Position	ToxoDB GT1 ID	ToxoDB annotation	Fold change RH-ERP/ GT1 (Microarray)	Possible involvement in *Toxoplasma* process
5′ UTR	TGGT1_041490	dihydrolipoamid dehydrogenase, putative	1.7	Growth
5′ UTR	TGGT1_027570	microneme protein, putative	1.6	Invasion
3′ UTR	TGGT1_114880	tRNA splicing 2′ phosphotransferase, putative	1.6	Translation
5′ UTR	TGGT1_090150	NBP2B protein, putative	1.6	Unknown
5′ UTR	TGGT1_073790	transporter, major facilitator family protein	-5.1	Growth
**B. Candidate genes consistently differentially expressed between RH-ERP and GT1**				
ToxoDB GT1 ID		ToxoDB annotation	Fold change RH-ERP/GT1 (Microarray)	Possible involvement in *Toxoplasma* process
TGGT1_098460		ankyrin repeat-containing protein	12.1	Transcriptional control
TGGT1_073210		hypothetical protein	5.7	Unknown
TGGT1_020630		hypothetical protein (contains glycosyltransferase 17 family)	5.7	Unknown
TGGT1_051960		ABC transporter transmembrane region domain-containing protein	3.0	Growth
TGGT1_030200		Peptidyl-tRNA hydrolase PTH2 domain-containing protein	2.3	Growth
TGGT1_000480		GRA12 homologue	2.0	Growth
TGGT1_126470		hypothetical protein	-2.1	Unknown
TGGT1_048210		rhoptry kinase family protein ROP38 (ROP38)	-3.7	Host cell modulation
TGGT1_047990		rhoptry kinase family protein ROP29 (ROP29)	-4.0	Host cell modulation
TGGT1_081480		zinc finger (CCCH type) motif-containing protein	-8.0	Transcriptional control
TGGT1_021770		microneme protein, putative	-9.2	Invasion
TGGT1_126670		rhoptry protein ROP8 (ROP8)	-11.3	Host cell modulation

**(A)** Candidate genes that have a SNP/indel present in 1000 bp region upstream of predicted ATG start or downstream of predicted end codon, and leading to expression differences of ≥1.5 fold in *Toxoplasma* arrays. **(B)** Candidate genes that are consistently differentially expressed between RH-ERP and GT1 using the three *Toxoplasma* array datasets, but not necessarily containing SNPs/indels in the 5′ or 3′ UTR regions.

### RH-ERP and GT1 GRA2 both complement Irgb6 coating but show differences in IRG mediated killing

As mentioned above, GRA2 was found to have a single polymorphism from glycine (GT1) to serine (RH-ERP) in our SNP analysis. Furthermore, previous work on *Toxoplasma* immune evasion mechanisms have shown that RH-ERP infected IFN-γ stimulated MEFs had decreased percentage of parasite vacuoles coated with Irgb6, an immunity related GTPase (IRG), compared to GT1 infected MEFs [21]. In addition, RHΔ*gra2* infected IFN-γ stimulated MEFs had increased percentage of parasite vacuoles coated with Irgb6 compared to RH-ERP infected MEFs. Thus, we examined the possible effects of the GRA2 polymorphism on Irgb6 coating and IRG evasion through complementation of RHΔ*gra2* with either RH-ERP *GRA2* or GT1 *GRA2*.

**Figure 1:**
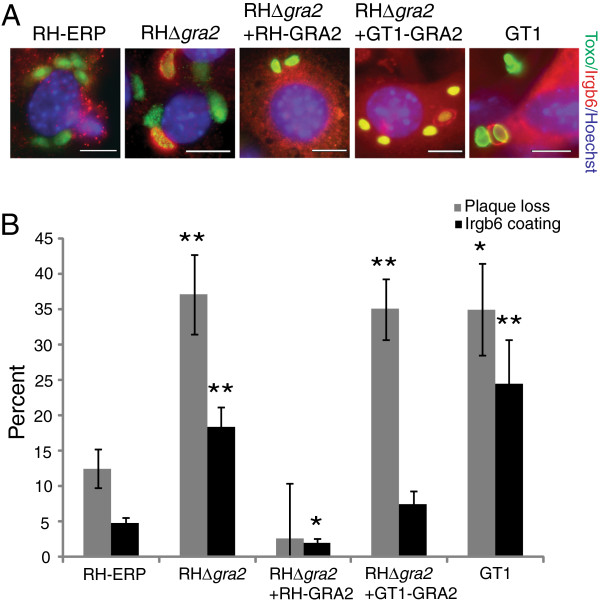
**Differences in complementation of plaque loss and Irgb6 coating between RH-ERP and GT1 GRA2. (A)** Mouse embryonic fibroblasts were stimulated with IFNγ for 24 hours, infected with RH-ERP, RHΔ*gra2*, RHΔ*gra2*+RH-ERP-GRA2, RHΔ*gra2*+GT1-GRA2 and GT1 for 1 hour and stained for Irgb6 (red), Hoechst (blue) and SAG1 (RH-ERP, RHΔ*gra2* and GT1) or TdTomato (complemented strains) (green). Scale bars represent 10 μm. **(B)** Quantification of Irgb6 localization on the parasite containing vacuole and percentage plaque loss after 4–7 days on IFNγ stimulated MEFs compared to unstimulated MEFs. Mean + SEM, of at least 4 independent experiments, * p-value < 0.05 and ** p-value < 0.01, Student’s *t*-test compared to RH-ERP infected MEFs.

As noted previously, there was a significant difference between RH-ERP and RHΔ*gra2* or GT1 in percentage of parasite vacuoles coated with Irgb6 (Figure 1A and 1B, *p*-value = 0.0004 (RHΔ*gra2*) and *p*-value = 0.002 (GT1), Student’s *t* test). Both RH-ERP GRA2 and GT1 GRA2 were able to complement Irgb6 coating, as both complemented strains had similar percentage parasite vacuoles coated with Irgb6 compared to RH-ERP. However, some *Toxoplasma* parasites are still able to survive, escape IRG coated vacuoles and invade a new cell, thus Irgb6 coating may not fully measure parasite killing [49]. Therefore, to measure IFN-γ mediated killing in MEFs, the relative number of parasite plaques that form 4–7 days on IFN-γ stimulated MEFs compared to unstimulated MEFs were measured (also referred to as plaque loss). Surprisingly, unlike Irgb6 coating, there were differences between the two complemented strains in the plaque loss assay (Figure 1B), though both complemented strains expressed similar levels of GRA2 (Additional file 2: Figure S1). The RH-ERP *GRA2* was able to complement the difference in plaque loss between RH-ERP and RHΔ*gra2*, with no significant difference in plaque loss between RH-ERP and RHΔ*gra2*+RH-ERP-GRA2 (*p*-value = 0.33, Student’s *t* test). However, the GT1 *GRA2* was unable to complement the difference in plaque loss, and there was still a significant difference in plaque loss between RH-ERP and RHΔ*gra2*+GT1-GRA2 (*p*-value = 0.008, Student’s *t* test), similar to the difference observed between RH-ERP and GT1 (*p*-value = 0.01, Student’s *t* test) (Figure 1B).

It is possible that the difference in complementation observed between RH-ERP GRA2 and GT1 GRA2 in the plaque assay is due to *Toxoplasma* killing in MEFs involving other IRG family members in addition to Irgb6. The different IRGs are known to play individual, non-redundant roles during infection, as mice lacking different IRGs exhibit susceptibility to *Toxoplasma* infection at different stages of infection [50]. Furthermore, Irgb6 coating is likely dependent on proper parasitophorous vacuole biogenesis, which is disrupted in RHΔ*gra2*[35], whereas plaque loss is likely not solely dependent on proper PV biogenesis.

### Identification and analysis of differentially expressed parasite genes among type I strains

In addition to polymorphisms, phenotypic differences between RH-ERP and GT1 can also be attributed to differential expression of parasite genes among the different type I strains. This differential expression could be due to two main reasons [51]. Firstly, polymorphisms in the *cis*-regulatory regions, such as the promoter or untranslated regions (UTRs), could lead to differences in transcription initiation, transcript stability or transcript decay. Secondly, polymorphic or differentially expressed transcriptional regulators, such as the Apetala 2 (AP2) transcription factors [52], could affect the expression levels of non-polymorphic genes. These transcriptional regulators can affect gene expression levels at different levels of RNA metabolism, such as controlling the formation of the exon junction complex, the export of mRNA through the nuclear complex and translation initiation [36,52,53].

Since sequence variation in promoter regions between strains could lead to differential gene expression, we determined whether any of the 1164 SNPs/indels we identified outside predicted gene coding regions were inside a promoter/5′ UTR (defined as 1000 bp upstream of predicted ATG start codon) or 3′ UTR (defined as 1000 bp downstream of predicted end codon). We found 133 SNPs/indels within the 5′ UTR of 110 genes and 143 SNPs/indels within the 3′ UTR of 106 genes. Of these 216 genes with 5′ or 3′ UTR SNPs/indels, microarray analyses showed that 43 genes were ≥1.5 fold differentially expressed between RH-ERP and GT1 (p-value = 0.07, hypergeometric test) (Additional file 1: Table S1).

We identified a putative dihydrolipoamid dehydrogenase (TGGT1_041490) with a 5′ UTR polymorphism, that was expressed 1.7 fold higher in RH-ERP compared to GT1 using array data, and was also expressed higher than in other canonical strains such as PRU and VEG (ToxoDB.org). Moreover, this same putative dihydrolipoamid dehydrogenase was expressed approximately 11 fold higher in RH-ERP intracellular parasites compared to extracellular parasite (Table 3A and Additional file 1: Table S1), which could indicate a possible role in intracellular growth. Dihydrolipoamide dehydrogenase serves as a component of several multifunctional complexes, such as pyruvate dehydrogenase, the glycine cleavage system and branched chain amino acid dehydrogenase complexes [54]. Thus, increased expression of this gene in RH-ERP could lead to differences in glycolytic metabolism, or resistance to reactive nitrogen intermediates. Interestingly, deletion of dihydrolipoamide dehydrogenase in an intracellular bacterial pathogen, *Mycobacterium tuberculosis*, causes a significant attenuation of virulence in mice [55].

Notably, there was a single ABC transporter (TGGT1_025370, also called TgABCG_107_[56]) with a 5′ UTR polymorphism, though it was only expressed 1.3 fold higher in RH-ERP compared to GT1 using array data. It was shown that cells transfected with TgABCG_107_ accumulated larger amounts of cholesterol in an ATP-dependent manner compared to untransfected cells, indicating a role for ABCG_107_ in lipid homeostasis in *Toxoplasma*[56]. In addition, we noted an ATP dependent helicase (TGGT1_113930) with a 5′ UTR polymorphism that was expressed 1.4 fold higher in RH-ERP compared to GT1 using array data (Additional file 1: Table S1). Thus, overexpression of these ATP dependent candidate genes identified could contribute to the enhanced growth of RH-ERP.

Interestingly, we identified an AP-2 transcription factor (AP2VIIA2; TGGT1_072850) with a 3′ UTR polymorphism, and is more highly expressed in M4 *in vitro* bradyzoites and *in vivo* cysts compared to *in vitro* tachyzoites [46] (Additional file 1: Table S1). 24 AP-2 transcription factors have cyclical expression profiles corresponding to the tachyzoite division cycle, whereas 11 AP-2 mRNAs are induced during *in vitro* bradyzoite differentiation (including AP2VIIA2) [52]. Therefore, this AP2 transcription factor could be involved in the regulation of genes required for bradyzoite differentiation and cyst formation.

Another reason for differential parasite gene expression could be polymorphic or differentially expressed trans-regulators, such as the AP2 transcription factors [52] that could regulate the expression of many non-polymorphic genes. Alternatively, differential expression of genes could be under epigenetic control, such as post translational modification of histone proteins or arginine methylation [57,58]. Thus, we investigated genes that were differentially expressed between RH-ERP, RH-JSR and GT1, regardless of SNPs in the coding or regulatory regions (Additional file 3: Table S2). We used expression datasets available from a previous published study [15], another independent dataset with parasite gene expression levels comparing RH-ERP and GT1 [22], and our own *Toxoplasma* arrays measuring parasite gene expression levels comparing RH-ERP, RH-JSR and GT1 (GSE44191). We analyzed for transcripts that were ≥1.5 fold differentially expressed between RH-ERP and GT1 across these three datasets, to identify genes that were consistently up or downregulated between RH-ERP and GT1. We identified 13 transcripts that had consistently increased and 13 transcripts that had consistently decreased expression in RH-ERP compared to GT1 across all three independent datasets respectively.

From the 13 consistently upregulated transcripts in RH-ERP compared to GT1 (Additional file 3: Table S2), we identified a putative ankyrin repeat containing protein (TGGT1_098460). This gene was highly expressed in RH-ERP in both array and RNA-seq datasets, but not in GT1 (Table 3B), and had lower expression in canonical strains such as PRU and VEG (ToxoDB.org). Ankyrin repeats mediate molecular recognition via protein-protein interactions [59], and proteins containing these repeats are involved in a large number of cellular functions, ranging from modulation of the NF-kB response to transcriptional regulation [60]. Interestingly, TgANK-1, a parasite protein containing ankyrin repeats, is induced upon bradyzoite differentiation using RH parasites, and localizes to the parasite cytosol [61]. Another gene that was consistently upregulated was a GRA12 homologue, and GRA12 co-localizes with GRA2 and interacts with GRA2 or GRA2-associated proteins [62]. Therefore, this gene could be associated with the increased growth rate of RH-ERP compared to GT1.

Of the 13 transcripts with increased expression in GT1 compared to RH-ERP, three encode for known rhoptry proteins, ROP8 (TGGT1_126670), ROP29 (TGGT1_047990) and ROP38 (TGGT1_048210) (Table 3C). ROP29 and ROP38 are part of a repeated gene family, and overexpression studies of ROP38 in the RH-ERP background showed that ROP38 has major effects on host gene expression [63]. Furthermore, as ROP8, ROP29 and ROP38 have predicted signal peptides, these rhoptry proteins might be involved in differential host modulation between RH-ERP and GT1. Several rhoptry proteins are known to be secreted into the host cells, where they play major roles in modulation of the host cell functions [64].

### Differentially modulated parasite pathways between type I strains

**Figure 2:**
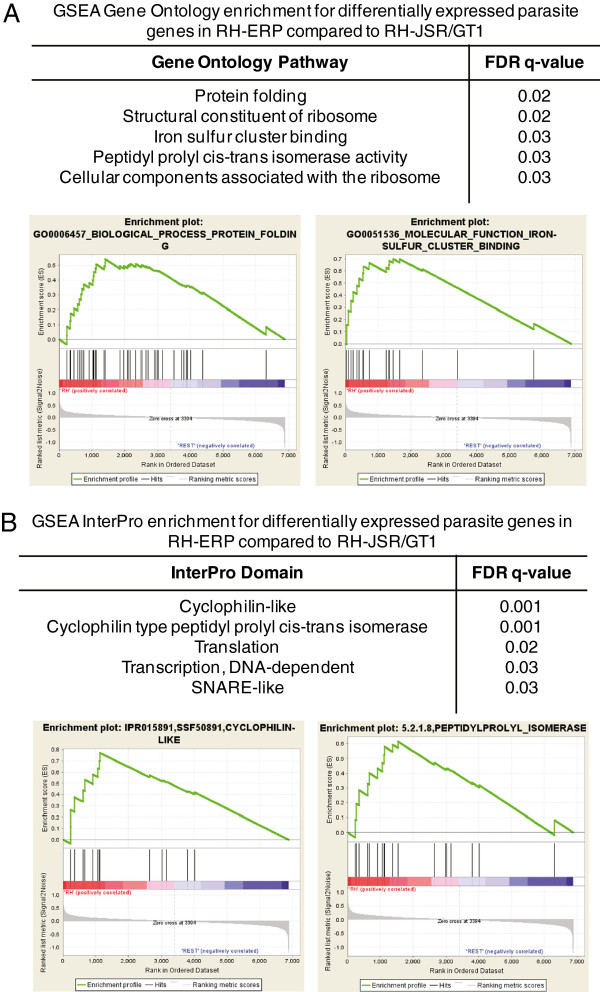
**Differential parasite gene expression between type I strains. (A)** The top five enriched Gene Ontology (GO) pathways using GSEA in differentially expressed parasite genes in RH-ERP compared to RH-JSR/GT1 and the corresponding GSEA diagrams, using *Toxoplasma* genesets with Gene Ontology annotations. The GSEA diagrams show the enrichment score (green line), which reflects the degree to which that particular gene set (header above) is overrepresented in the differentially expressed genes between RH-ERP and RH-JSR/GT1 (ranked by their differential expression values). The middle portion of the diagram shows where the members of the particular gene set appear in the ranked gene list. The bottom portion of the diagram shows the value of the ranking metric, which measures the correlation of a gene with upregulation (positive value) or downregulation (negative value) in RH-ERP compared to RH-JSR/GT1. **(B)** The top five enriched InterPro domain using GSEA in differentially expressed parasite genes in RH-ERP compared to RH-JSR/GT1 and the corresponding GSEA diagrams, using *Toxoplasma* genesets with InterPro annotations.

In addition, we analyzed whether the differentially expressed genes between RH-ERP, RH-JSR and GT1 across the three independent datasets were enriched in annotated biological functions [65]. We focused on functional enrichment of parasite genes differentially expressed in RH-ERP compared to RH-JSR and GT1, using our own arrays and expression data from previous studies [15,22]. There were 15 Gene Ontology (GO) and 25 InterPro genesets that were significantly enriched in RH-ERP compared to RH-JSR/GT1 (false discovery rate (FDR) < 0.10). Parasite genes differentially expressed in RH-ERP compared to RH-JSR/GT1 were enriched in GO processes such as transcription, translation, protein folding and iron-sulfur cluster binding (Figure 2A and Additional file 4: Table S3). Similarly, parasite genes differentially expressed in RH-ERP compared to RH-JSR/GT1 were enriched in InterPro domains such as peptidyl-prolyl cis-trans isomerases, cyclophilin-like and DNA-dependent RNA polymerases (Figure 2B and Additional file 4: Table S3). This is in accord with observations that RH-ERP has a higher growth rate, likely requiring increased transcription and translation, and increased extracellular viability [15], likely requiring expression of stress proteins to survive extracellular stress.

### Differential modulation of host pathways between type I strains

**Figure 3:**
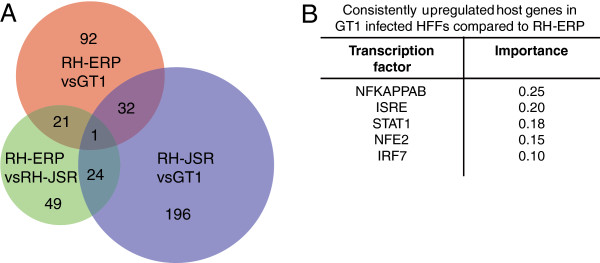
**Differential host gene expression between type I strains.** HFFs were infected for 24 hours with RH-ERP, RH-JSR and GT1 and host gene expression was analyzed using microarrays. Three arrays were done per strain for RH-ERP and RH-JSR, and two arrays were done for GT1. **(A)** Venn diagram showing the number of host genes that were consistently differentially expressed ≥1.5 fold or more for each pairwise comparison between type I strains. **(B)** Top five enriched known transcription factors from DiRE analysis of consistently upregulated host genes in GT1 infection compared to RH-ERP.

Because some of the differentially expressed or polymorphic *Toxoplasma* genes, such as ROP38, might be involved in the modulation of host cell signaling pathways [63], we determined whether different type I strains differ in their ability to modulate the host cell response. To do this, we infected HFFs with RH-ERP, RH-JSR and GT1 for 24 hours and determined host gene expression profiles using microarrays. We identified 146, 95, and 253 host transcripts that were consistently up/downregulated ≥1.5 fold in every experiment comparing RH-ERP against GT1, RH-ERP against RH-JSR and RH-JSR against GT1 respectively (Figure 3A and Additional file 5: Table S4). As ROP38 was differentially expressed between RH-ERP and GT1, and has major effects on host gene expression, we wanted to determine the effects of ROP38 in differentially expressed host transcripts between RH-ERP and GT1. To do this, we used expression datasets available from a previous study that compared host gene expression after infection with RH-ERP or RH-ERP overexpressing ROP38 [63]. From the 146 host transcripts that were consistently differentially expressed between RH-ERP and GT1, 14 were also ≥1.5 fold differentially expressed between RH and RH overexpressing ROP38 (Additional file 5: Table S4). Thus, these 14 genes which are differentially expressed between RH-ERP and GT1 could be due to ROP38 expression differences.

We subsequently determined if the promoters of the consistently differentially regulated host genes were enriched for specific transcription factor binding sites (TFBS) [30,31]. We noted an NF-κB signature in the promoter of host genes that were upregulated ≥1.5 fold in GT1 compared to RH-ERP (Figure 3B), and also noted that type I interferon and STAT1 signatures were present in host genes upregulated in HFFs infected with GT1 compared to RH-ERP. In addition, we observed STAT6 enrichment in the promoter of host genes that were consistently upregulated in RH-JSR compared to RH-ERP (Additional file 6: Table S5).

### NF-κB activation and induction of IL-12p40 secretion upon infection are differentially modulated by type I strains and are dependent on GRA15

**Figure 4:**
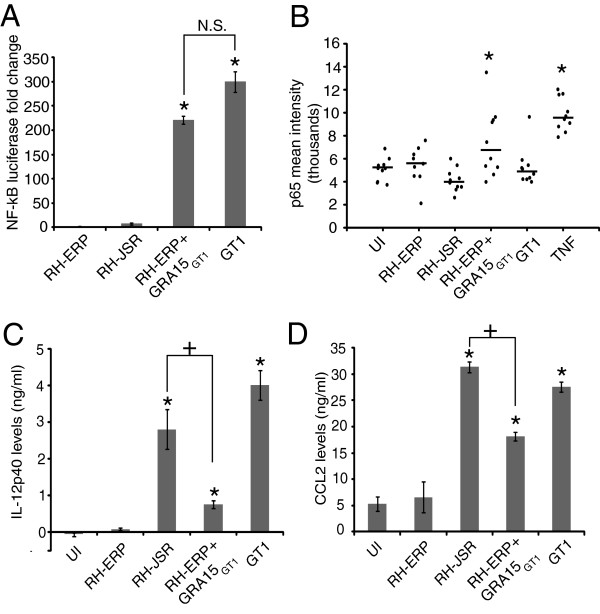
**Type I strains differentially activate NF-kB nuclear translocation, and is dependent on GRA15. (A)** HEK293T NF-kB reporter cells were infected with RH-ERP, RH-JSR, RH+GRA15_GT1_ and GT1. For each strain, the luciferase reading after 24 hours infection was taken. The graph shows averages from three independent experiments, with levels representing fold change of NF-kB luciferase readings normalized to uninfected, unstimulated control cells, and the error bars represent the standard error. Mean + SEM, of three experiments, * *p*-value < 0.01, N.S. not significant, Student’s *t*-test. **(B)** HFFs were infected for 24 h with RH-ERP, RH-JSR, GT1 and RH+GRA15_GT1_ transgenic, uninfected cells were stimulated with TNF-alpha for 1 h (TNF), or left unstimulated and uninfected (UI). Cells were fixed, probed with p65 antibody and mean nuclear staining was quantified, the error bars represent the standard deviation, * *p*-value < 0.05, Student’s *t*-test. Quantification shown is representative of three independent experiments. **(C)** IL12-p40 and **(D)** CCL2 levels were measured in supernatants from C57BL/6 bone marrow derived macrophages infected for 24 hours with RH-ERP, RH-JSR, RH+GRA15_GT1_ transgenic and GT1. The graphs shown are representative of three independent experiments for IL-12p40 and two independent experiments for CCL2, and the error bars represent the standard deviation, * *p*-value < 0.01, Student’s *t*-test between strain and RH-ERP infected BMMs, + *p*-value < 0.01, Student’s *t*-test between the two conditions indicated.

Previous studies have shown that type II strains activate NF-κB to a much higher level compared to types I and III, and this difference is due to polymorphisms in the dense granule protein, GRA15 [23]. It was noted that high levels of nuclear translocation of the p65 subunit of NF-κB were present in host cells infected with several type II strains (ME49, PRU, DAG and Beverly), while host cells infected with RH-ERP and GT1 had much lower levels of p65 translocation [23]. Our host transcriptional profile analysis across the type I strains indicated that there is an enrichment in NF-κB binding sites in the promoters of host genes that are more highly induced by GT1 infection compared to RH-ERP (Figure 3B). To validate this analysis, HEK293T NF-κB reporter cells with NF-κB binding sites driving the expression of GFP and luciferase were infected with RH-ERP, RH-JSR and GT1 for 24 hours and assayed for NF-κB dependent luciferase activity (Figure 4A). In accord with the transcriptional profiling analysis, GT1 infection induced much higher NF-κB mediated luciferase activity compared to RH-ERP (*p*-value = 0.001, Student’s *t* test), although this induction was still 2–3 fold lower compared to cells infected with Pru, a type II strain (data not shown).

We then investigated whether GRA15 could explain the differences in NF-κB activation between the type I strains, since our SNP analysis indicated an indel in GRA15 when comparing RH-ERP and GT1 (Additional file 1: Table S1). Moreover, RH-ERP was shown to contain a frameshift deletion compared to GT1, leading to a nonfunctional GRA15 in RH-ERP [23]. Because RH-JSR also does not induce NF-κB activation, we sequenced *GRA15* from RH-JSR and, surprisingly, we found that RH-JSR contains a frameshift insertion at base 734. This mutation is independent from the RH-ERP frameshift deletion at base 872. The RH-JSR insertion causes a mutation of a stretch of 34 amino acids followed by a premature stop codon (Additional file 7: Figure S2). Thus, both RH-ERP and RH-JSR contain truncated GRA15 proteins of 312 and 278 amino acids respectively, while GT1 has the full length protein of 635 amino acids. GRA15 was observed to affect parasite growth both *in vitro* and *in vivo*, as RH-ERP expressing GRA15_II_ has reduced plaque size in HFFs and reduced parasite burden in mice [23]. Thus, there could have been a selective pressure for a nonfunctional GRA15 in RH-ERP and RH-JSR, allowing for increased parasite burden in the host or faster replication *in vitro*.

Since RH-ERP provides a null genetic background with respect to GRA15, we generated transgenic RH-ERP parasites overexpressing GRA15 from GT1 and assayed for NF-κB mediated luciferase activity using the same NF-κB reporter cell line described above (Figure 4A). The NF-κB reporter luciferase activity after infection with RH-ERP+GRA15_GT1_ was significantly higher than the activity after infection with RH-ERP (*p*-value = 0.0001, Student’s *t* test). This data supports the hypothesis that differences in NF-κB activation among type I strains are due to GRA15. In addition, the NF-κB family of transcription factors consist of five members, p65, c-REL, REL-B, p50 and p52 [66], and the NF-κB reporter contains four repeated, canonical NF-κB binding sites which can be bound by the different NF-κB subunits. Previous studies showed p65 nuclear localization in host cells infected with type II strains [23] and we wanted to determine whether p65 nuclear translocation could be responsible for differential NF-κB activation observed in host cells infected by GT1 compared to RH-ERP. HFFs were infected with RH-ERP, RH-JSR, GT1 or transgenic RH-ERP+GRA15_GT1_, and nuclear localization of p65 was examined by immunofluorescence (Figure 4B). There was significant nuclear translocation of p65 in cells infected with transgenic RH-ERP+GRA15_GT1_ parasites compared to uninfected cells (*p*-value = 0.02, Student’s *t* test), but no significant p65 nuclear translocation was observed in cells infected with RH-ERP and GT1 (*p*-value > 0.05, Student’s *t* test). It is possible that the GRA15 in GT1 activates a different NF-κB subunit from p65, such as c-REL or p50, but transgenic high overexpression of GRA15_GT1_ in RH-ERP activates p65 in addition to other subunits. It is also possible that there are other GT1 genes that could have an inhibitory effect on p65 translocation.

GRA15 from type II strains (GRA15_II_) was also shown to affect levels of IL-12p40, a NF-κB dependent cytokine, by infected macrophages *in vitro*[23], and regulation of IL12-p40 production has been linked to NF-κB activation [67]. Thus, we investigated whether type I strains differ in their ability to induce secretion of IL-12p40 and CCL2, another NF-κB dependent cytokine, and examined whether GRA15 from GT1 played a role in these differences. GT1 infection of C57BL/6 bone marrow derived macrophages resulted in secretion of higher levels of both IL-12p40 and CCL2 in culture supernatants compared to RH-ERP infection (Figures 4C and 4D, *p*-value = 7.5×10^-5^ (IL-12p40) and *p*-value = 0.0003 (CCL2), Student’s *t* test). This phenotype can be partially attributed to GRA15, as macrophages infected with RH-ERP+GRA15_GT1_ induced higher levels of IL-12p40 and CCL2 compared to RH-ERP (Figure 4C and 4D, *p*-value = 0.0006 (IL-12p40) and *p*-value = 0.003 (CCL2), Student’s *t*-test). However, GRA15_GT1_ in the RH+GRA15_GT1_ background was not sufficient to increase secretion of IL12-p40 by infected macrophages to levels comparable to those observed in macrophages infected with GT1. Moreover, infection with RH-JSR, which has a nonfunctional GRA15, induced IL12-p40 to levels higher than infection with RH-ERP+GRA15_GT1_ (*p*-value = 0.003 (IL12-p40) and *p*-value = 5.9x10^-5^ (CCL2), Student’s *t*-test). Therefore, it is likely that other *Toxoplasma* genes contribute to induction of IL-12p40 and CCL2 production by infected macrophages, especially given that the regulation of IL12-p40 is more complex than sole control by NF-κB [68].

### Localization of p-IκBα at the PVM is strain specific, independent of NF-kB activation and partially dependent on GRA2

In the canonical NF-κB activation pathway, IκBα normally inhibits NF-κB translocation and sequesters NF-κB in the cytoplasm. However, upon stimulation, IκBα is phosphorylated and subsequently targeted for proteasomal degradation via ubiquitination, exposing the nuclear localization signal in NF-κB and allowing nuclear localization to occur [66]. It has been previously reported that phospho-IκBα (p-IκBα) is localized to the PVM in RH-ERP infected mouse embryonic fibroblasts (MEFs), and this has been linked to NF-κB activation [69,70]. However, there was little or no p-IκBα localization at the PVM in cells infected with type II and type III strains [23]. Moreover, localization of p-IκBα was observed using RH-ERP, and it is currently unknown whether the same phenomenon holds true for other type I strains.

**Figure 5:**
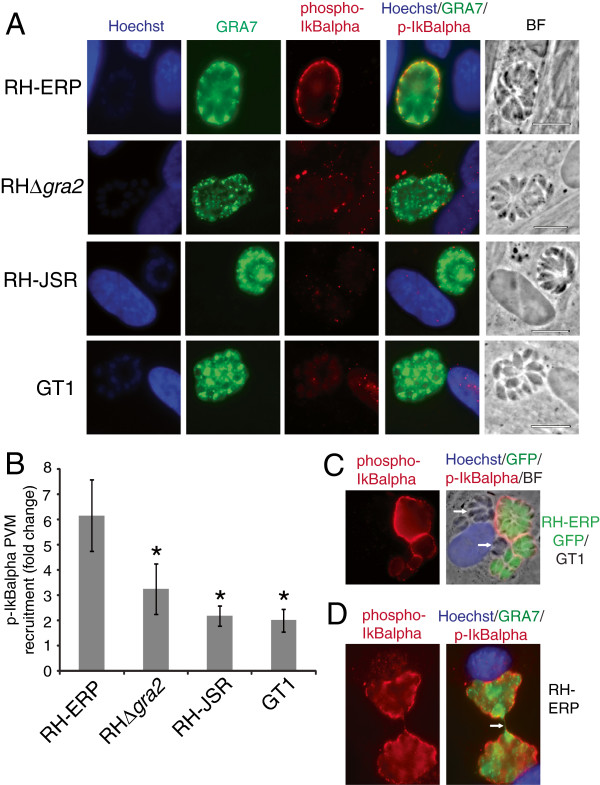
**p-IκBα localizes to the parasitophorous vacuolar membrane of RH-ERP, but not RH-JSR or GT1, and is partially dependent on GRA2. (A)** Human foreskin fibroblasts were infected with type I strains for 24 hours, fixed with formaldehyde and stained with p-IκBα (red), GRA7 (green) and Hoechst (blue). Pictures are representative of at least three experiments. **(B)** Quantification of p-IκBα recruited to the PVM of RH-ERP, RH∆*gra2*, RH-JSR and GT1. The intensity of p-IκBα recruited to the PVM was quantified in at least 5 cells per condition. The graph shows the average from three independent experiments, with levels showing average p-IκBα recruitment quantification, and the error bars represent standard error. * *p*-value < 0.01, Student’s *t* test. **(C)** HFFs were infected with RH-GFP and GT1 for 24 hours, fixed with formaldehyde and stained with p-IκBα (red), GRA7 (green) and Hoechst (blue). Arrows point to GT1 vacuoles. **(D)** Mouse embryonic fibroblasts were infected with RH-ERP for 30 hours, fixed with formaldehyde and stained with p-IκBα (red), GRA7 (green) and Hoechst (blue). Arrows point to the intervacuolar PVM extension.

Thus, to determine whether p-IκBα is also redirected to the PVM in cells infected with other type I strains, immunofluorescence (IF) with antibodies against p-IκBα was performed in HFFs infected with RH-ERP, RH-JSR and GT1. In agreement with previous studies, p-IκBα localized to the PVM of RH-ERP, but there was much less accumulation of p-IκBα at the PVM in HFFs infected with other type I strains (Figure 5A). Quantification of the intensity of p-IκBα around the PVM confirmed that vacuoles containing RH-ERP recruited significantly more p-IκBα than vacuoles with RH-JSR or GT1 (Figure 5B, *p*-value = 0.009 (RH-JSR) and 0.008 (GT1), Student’s *t*-test). It is unlikely that NF-κB activation levels are correlated with the level of p-IκBα recruitment around the PVM, given previous data that type II strains activate NF-κB in infected host cells but do not induce accumulation of p-IκBα to the PVM [23]. This is further supported by the observed differences in type I strains, since neither RH-ERP nor RH-JSR activate NF-κB (Figure 4A) but RH-ERP induces strong accumulation of p-IκBα at the PVM. In addition, localization of p-IκBα was similar in HFFs infected with RH-ERP+GRA15_GT1_ compared to RH-ERP infected HFFs (data not shown), even though RH-ERP+GRA15_GT1_ activates NF-κB, which further supports that NF-κB activation is unrelated to accumulation of p-IκBα on the PVM.

To determine whether accumulation of p-IκBα at the PVM could be due to a soluble factor secreted by RH-ERP, immunofluorescence was performed in cells co-infected with both RH-ERP and GT1. Recruitment of p-IκBα to the PVM was observed only in vacuoles containing RH-ERP (GFP^+^), whereas little or no p-IκBα was recruited to the GT1 PVM (Figure 5C), implying that p-IκBα translocation is not induced by soluble secreted parasite factors. We also tested the effect of ROP16, a known secreted rhoptry kinase, on p-IκBα recruitment, and saw no observable differences between RH-ERP and RHΔ*rop16* (Additional file 8: Figure S3). We also observed that p-IκBα colocalized with GRA7 at PVM extensions (Figure 5D) where another dense granule protein, GRA14, has been described to be trafficked to [71]. Furthermore, as mentioned before, GRA2 is known to affect the vesicular network in the PV and PVM [72,73] and we identified that RH-ERP GRA2 differs in one amino acid from RH-JSR/GT1 GRA2 (Additional file 1: Table S1).

Thus, we examined whether dense granule proteins had an effect on p-IκBα recruitment. Even though there were no observable differences in PVM localization of p-IκBα between RH-ERP and RHΔ*gra15* (Additional file 8: Figure S3), there was observed reduction of p-IκBα accumulation at the PVM in cells infected with RHΔ*gra2* (Figure 5B). Since the vacuoles formed by the RHΔ*gra2* exhibit disruptions in the intravacuolar network [35], lack of p-IκBα recruitment to the PVM could be due to disruption of parasitophorous vacuole biogenesis. Alternatively, as GRA2 has been found in complexes with other dense granule proteins [74], these GRA proteins could be directly interacting with host IκBα.

In addition, complementation of RHΔ*gra2* with either RH-ERP GRA2 or GT1 GRA2 partially restored the p-IκBα localization phenotype, with increased PVM recruitment of p-IκBα present in both complemented strains compared to RHΔ*gra2* (Additional file 9: Figure S4A). Quantification of the intensity of p-IκBα around the PVM confirmed that both complemented strains did not have significantly different PVM recruitment of p-IκBα compared to RH-ERP (Additional file 4: Figure S4B, *p*-value = 0.45 (complemented RH-ERP GRA2) and *p*-value = 0.58 (complemented GT1 GRA2), Student’s *t*-test). This suggests that although GRA2 expression is necessary for full accumulation of p-IκBα to the PVM, it is likely due to proper parasitophorous vacuole biogenesis rather than the specific polymorphism present in GRA2 between RH-ERP and GT1.

## Conclusions

In summary, through comparative genome and gene expression analysis, we identified a list of candidate genes that could be responsible for the phenotypic differences between different type I strains. We show that polymorphisms in *GRA2* and *GRA15* determine type I strain differences in survival in IFN-γ stimulated cells and activation of NF-κB, respectively. Future experiments will focus on the contribution of the individual genes to the increased growth rate, higher extracellular viability and loss of orally infectious cyst formation ability in RH-ERP. Thus, these identified parasite genes and differentially modulated host pathways could lead to design of new parasite targets relevant to *Toxoplasma* pathogenesis and chronic infection. However, it is likely that many of these phenotypes are affected by a combination of SNPs/indels and/or expression differences across multiple parasite genes, rather than being controlled by individual or a few genes.

### Availability of supporting data

The data sets supporting the results of this article are available in the GEO repository as a GEO SuperSeries, GSE44246, http://www.ncbi.nlm.nih.gov/geo/query/acc.cgi?acc=GSE44246.

## Abbreviations

EGR: Early growth response factor; FDR: False discovery rate; GRA: Dense granule protein; HFF: Human foreskin fibroblast; IRG: Immune related GTPase; Indels: Insertions/deletions; IκBα: Inhibitor of Nuclear factor kappa-light-chain-enhancer of activated B cells; MEF: Mouse embryonic fibroblast; NF-κB: Nuclear factor kappa-light-chain-enhancer of activated B cells; PVM: Parasitophorous vacuole membrane; ROP: Rhoptry protein; SNP: Single nucleotide polymorphism; SRF: Serum response factor; UTR: Untranslated region.

## Competing interests

The authors declare that they have no competing interests.

## Authors’ contributions

JPJS and NY analyzed the sequencing and parasite/host gene expression data. NY performed luciferase assays, immunoassays and p-IκBα immunofluorescence assays. AF, GTM and MJG performed, processed and annotated the sequencing data between RH-ERP and GT1. WN made the GRA2 complemented strains and performed plaque assays and IRG evasion. MM contributed RNA-seq data for RH-ERP and GT1. DL and LJ made the GRA15_GT1_ complemented strain. JPJS and NY conceived the study and wrote the manuscript. All authors provided comments on the paper, read and approved the final manuscript.

## Supplementary Material

Additional file 1: Table S1 List of single nucleotide polymorphisms and insertions/deletions present between RH-ERP and GT1 through whole genome sequencing. A total of 1,394 SNPs/indels were identified between RH-ERP and GT1, with 230 SNPs/indels in predicted gene coding regions, 484 SNPs/indels in predicted genes (outside coding regions) and 680 SNPs/indels outside predicted genes. SNPs/indels with gene IDs have been annotated with ToxoDBv8 descriptions, *Toxoplasma* arrays for parasite gene expression between RH-ERP, RH-JSR and GT1 [15,22] and RNA-seq data for RH-ERP and GT1. SNPs/indels have also been annotated whether they are present in 1000 bp upstream of predicted ATG start (5′UTR) or 1000 bp downstream of predicted end codon (3′UTR). For SNP/indels in 5′ or 3′ UTR, the corresponding genes have been annotated with *Toxoplasma* array parasite gene expression levels and RNAseq data. Click here for file

Additional file 2: Figure S1 Expression and localization of GRA2 in the RH∆*gra2* complemented strains. (A) Immunofluorescence of HA (red) in RH∆*gra2* complemented with RH-ERP-GRA2-HA or GT1-GRA2-HA parasites, co-stained with Hoechst (blue) and TdTomato (green). (B) Western blot for HA (top) and SAG1 (bottom) comparing HA expression of RH∆*gra2* complemented with RH-ERP-GRA2-HA or GT1-GRA2-HA. Click here for file

Additional file 3: Table S2 Consistently differentially expressed *Toxoplasma* genes between RH-ERP, RH-JSR and GT1. Parasite transcripts which were consistently differentially expressed ≥1.5 fold between RH-ERP and GT1 in the same direction in two previous studies [15,22] and arrays used in this study were identified. These consistently differentially expressed genes were annotated with whether they had increased or decreased expression in RH-ERP compared to GT1, ToxoDBv8 description, *Toxoplasma* array parasite gene expression levels and RNAseq data for RH-ERP and GT1. Click here for file

Additional file 4: Table S3 Differentially modulated *Toxoplasma* pathways present in RH-ERP compared to RH-JSR and GT1. Parasite transcript expression in RH-ERP, RH-JSR and GT1 in two previous studies [15,22] and in this study were analyzed for enrichment in any one strain using GSEA. Enriched parasite pathways in RH-ERP compared to RH-JSR/GT1, using GO and InterPro annotations, which had a false discovery rate < 0.1 were identified. Click here for file

Additional file 5: Table S4 Consistently differentially expressed host genes between RH-ERP, RH-JSR and GT1. Human foreskin fibroblasts were infected for 24 hours with RH-ERP, RH-JSR and GT1, and host gene expression was determined through microarrays. Host transcripts which were consistently differentially expressed ≥1.5 fold between RH-ERP, RH-JSR and GT1 in each pairwise comparison were identified. Consistently differentially expressed host transcripts which were also ≥1.5 fold between RH and RH overexpressing ROP38 from a previous study were also identified [63]. Transcripts were annotated with gene description, strand type, GO term function, Gene Symbol and RefSeq IDs from the HG18 database. Click here for file

Additional file 6: Table S5 DiRE transcription factor binding site analysis of consistently differentially regulated host genes between type I strains. Host genes which were consistently differentially expressed ≥ 1.5 fold in each type I strain in a pairwise comparison were subject to TFBS analysis by DiRE. The top 10 TFs (with importance value > 0.1) associated with upregulated or downregulated host genes for a particular pairwise comparison are shown. Click here for file

Additional file 7: Figure S2 Differences in GRA15 sequence between RH-ERP, RH-JSR and GT1. RH-JSR and RH-ERP contain indels at position 734 and 872 respectively, which lead to independent frameshifts and early stop codons in the GRA15 protein. Dots represent consensus with the GT1 GRA15 sequence, dashes represent missing amino acids in RH-ERP or RH-JSR GRA15 compared to GT1 GRA15, red indicate amino acids different in RH-ERP from GT1 and purple indicate amino acids different in RH-JSR from GT1. Click here for file

Additional file 8: Figure S3 No differences in PVM localization of p-IκBα present between RH-ERP, RH∆*rop16* and RH∆*gra15*. Human foreskin fibroblasts were infected with RH-ERP and knockout strains at intended MOI 1 for 30 hours, fixed with formaldehyde and stained with p-IκBα (red), GRA7 (green) and Hoechst (blue). Pictures are representative of at least two experiments. Click here for file

Additional file 9: Figure S4 Partial restoration in PVM localization of p-IκBα present in RH∆GRA2 complemented with either RH-ERP GRA2 or GT1 GRA2. (A) Human foreskin fibroblasts were infected with RH∆*gra2*, RH∆*gra2* complemented with either RH-ERP GRA2 or GT1 GRA2 for 30 hours, fixed with methanol and stained with p-IκBα (red), GRA7 (green) and Hoechst (blue). Pictures are representative of three experiments. (B) Quantification of p-IκBα recruited to the PVM of RH-ERP, RH∆*gra2* and RH∆*gra2* complemented with either RH-ERP GRA2 or GT1 GRA2. The intensity of p-IκBα recruited to the PVM was quantified in at least 5 cells per condition. The graph shows the average from three independent experiments, with levels showing average p-IκBα recruitment quantification, and the error bars represent standard error. * *p*-value < 0.01, Student’s *t* test. Click here for file
