# Supplementary figures and images for: Genetic basis for phenotypic differences between different Toxoplasma gondii type I strains
Source: BMC Genomics. 2013 Jul 10;14:467. doi: 10.1186/1471-2164-14-467 (PMC3710486; doi:10.1186/1471-2164-14-467)

# Supplementary Figure 1.

A

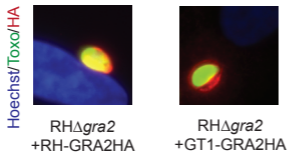

B

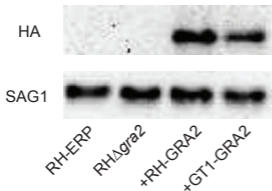

Supplement: Additional file 2: Figure S1 — Expression and localization of GRA2 in the RH∆gra2 complemented strains. (A) Immunofluorescence of HA (red) in RH∆gra2 complemented with RH-ERP-GRA2-HA or GT1-GRA2-HA parasites, co-stained with Hoechst (blue) and TdTomato (green). (B) Western blot for HA (top) and SAG1 (bottom) comparing HA expression of RH∆gra2 complemented with RH-ERP-GRA2-HA or GT1-GRA2-HA. [file 1471-2164-14-467-S2.pdf]

# Supplementary Figure 3.

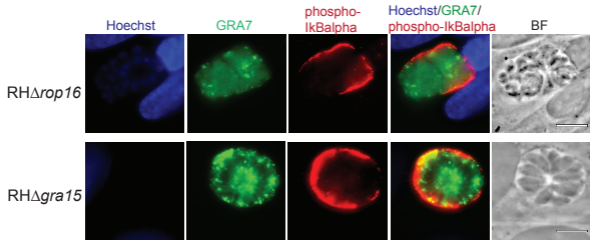

Supplement: Additional file 8: Figure S3 — No differences in PVM localization of p-IκBα present between RH-ERP, RH∆rop16 and RH∆gra15. Human foreskin fibroblasts were infected with RH-ERP and knockout strains at intended MOI 1 for 30 hours, fixed with formaldehyde and stained with p-IκBα (red), GRA7 (green) and Hoechst (blue). Pictures are representative of at least two experiments. [file 1471-2164-14-467-S8.pdf]

# Supplementary Figure 4.

**A**

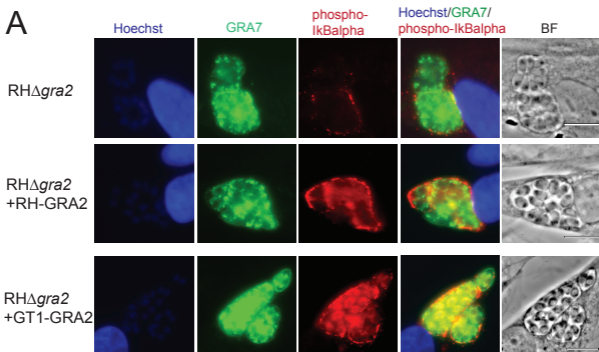

**B**

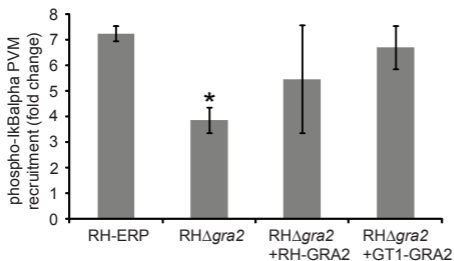

Supplement: Additional file 9: Figure S4 — Partial restoration in PVM localization of p-IκBα present in RH∆GRA2 complemented with either RH-ERP GRA2 or GT1 GRA2. (A) Human foreskin fibroblasts were infected with RH∆gra2, RH∆gra2 complemented with either RH-ERP GRA2 or GT1 GRA2 for 30 hours, fixed with methanol and stained with p-IκBα (red), GRA7 (green) and Hoechst (blue). Pictures are representative of three experiments. (B) Quantification of p-IκBα recruited to the PVM of RH-ERP, RH∆gra2 and RH∆gra2 complemented with either RH-ERP GRA2 or GT1 GRA2. The intensity of p-IκBα recruited to the PVM was quantified in at least 5 cells per condition. The graph shows the average from three independent experiments, with levels showing average p-IκBα recruitment quantification, and the error bars represent standard error. * p-value < 0.01, Student’s t test. [file 1471-2164-14-467-S9.pdf]
