# Supplementary material for: Genetic basis for phenotypic differences between different Toxoplasma gondii type I strains
Source: BMC Genomics. 2013 Jul 10;14:467. doi: 10.1186/1471-2164-14-467 (PMC3710486; doi:10.1186/1471-2164-14-467)
Supplement: Additional file 7: Figure S2 — Differences in GRA15 sequence between RH-ERP, RH-JSR and GT1. RH-JSR and RH-ERP contain indels at position 734 and 872 respectively, which lead to independent frameshifts and early stop codons in the GRA15 protein. Dots represent consensus with the GT1 GRA15 sequence, dashes represent missing amino acids in RH-ERP or RH-JSR GRA15 compared to GT1 GRA15, red indicate amino acids different in RH-ERP from GT1 and purple indicate amino acids different in RH-JSR from GT1. [file 1471-2164-14-467-S7.pdf]

Supplementary Figure 2.

```
GT1_GRA15       MVTTTTPTPP PGAPAVVPIF DVVYQLNPHV FRSRFSRRNR ARRVVSSKSR SIIRWLGYLT
RH-ERP_GRA15    .......... .......... .......... .......... .......... ..........
RH-JSR_GRA15    .......... .......... .......... .......... .......... ..........

GT1_GRA15       VLAAVILLGA YAVRRLSRDL SDSVRETRRG RRITGSVPPG TTRPRSESCT GTQVDGGCGA
RH-ERP_GRA15    .......... .......... .......... .......... .......... ..........
RH-JSR_GRA15    .......... .......... .......... .......... .......... ..........

GT1_GRA15       DTSTDGKSES EQTENGEDSR FSTRTPIHVT ASTSPFATRK AAEERSSSPR DRKVPEGAQL
RH-ERP_GRA15    .......... .......... .......... .......... .......... ..........
RH-JSR_GRA15    .......... .......... .......... .......... .......... ..........

GT1_GRA15       PTSSTPHAQR KDSGSDSRNP STLIPSPGTN TFNMNFYIIG AGSSALDFIF PHTPDAQATV
RH-ERP_GRA15    .......... .......... .......... .......... .......... ..........
RH-JSR_GRA15    .......... .......... .......... .......... .......... ..........

GT1_GRA15       VSPPRSAAAA PTVETVPRVR TYSTPTTLTL PTAPATATSN HMHASATPSP PERPQNFR.G
RH-ERP_GRA15    .......... .......... .......... .......... .......... RNVLKTSVGD
RH-JSR_GRA15    ....PQRGGR TNCRNSSKGS HLLDTNNINP TNGTSDRH-- ---------- ----------

GT1_GRA15       LMRQNGMVE. TSLTTTEAGM PAPLQSPQHI ETEARLTYSN HLKSPHTPET PTVHSIDPVV
RH-ERP_GRA15    SCGKTAWLRG HR-------- ---------- ---------- ---------- ----------
RH-JSR_GRA15    ---------- ---------- ---------- ---------- ---------- ----------

GT1_GRA15       GTSGHSVAVG SQSPAGGPPT DSRTPAALTP TSSSFSHADS LETSEHPQSG PSLHPLISGI
RH-ERP_GRA15    ---------- ---------- ---------- ---------- ---------- ----------
RH-JSR_GRA15    ---------- ---------- ---------- ---------- ---------- ----------

GT1_GRA15       QDAVQSQLPL SQQETLPVVE NATFFGPQQT PPWMDETAAA AIPLAPSQPG SRTQPISSPH
RH-ERP_GRA15    ---------- ---------- ---------- ---------- ---------- ----------
RH-JSR_GRA15    ---------- ---------- ---------- ---------- ---------- ----------

GT1_GRA15       TLLPLSGGVS AVPGPPRTEN PRQPQVPGEN SYYSVPTEPY PAQDMSPLIR GTHSQTETVE
RH-ERP_GRA15    ---------- ---------- ---------- ---------- ---------- ----------
RH-JSR_GRA15    ---------- ---------- ---------- ---------- ---------- ----------

GT1_GRA15       CGVNASSEGL AAGAPSSKSA ENAQTGQGAG KSLLPVFLHP QEQSPHSMPT LGAGRFGSGE
RH-ERP_GRA15    ---------- ---------- ---------- ---------- ---------- ----------
RH-JSR_GRA15    ---------- ---------- ---------- ---------- ---------- ----------

GT1_GRA15       LQRTISDPGP QRAGATQADG IGAGGPRDTQ SAVTP-
RH-ERP_GRA15    ---------- ---------- ---------- ------
RH-JSR_GRA15    ---------- ---------- ---------- ------
```
